# Persuasive Apps for Sustainable Waste Management: A Comparative Systematic Evaluation of Behavior Change Strategies and State-of-the-Art

**DOI:** 10.3389/frai.2021.748454

**Published:** 2021-12-09

**Authors:** Makuochi Nkwo, Banuchitra Suruliraj, Rita Orji

**Affiliations:** ^1^ Department of Computer Science, Ebonyi State University, Abakaliki, Nigeria; ^2^ Faculty of Computer Science, Dalhousie University, Halifax, NS, Canada

**Keywords:** sustainability, waste management, mobile apps, persuasive strategies, behavior change, systematic review

## Abstract

With the proliferation of ubiquitous computing and mobile technologies, mobile apps are tailored to support users to perform target behaviors in various domains, including a sustainable future. This article provides a systematic evaluation of mobile apps for sustainable waste management to deconstruct and compare the persuasive strategies employed and their implementations. Specifically, it targeted apps that support various sustainable waste management activities such as personal tracking, recycling, conference management, data collection, food waste management, do-it-yourself (DIY) projects, games, etc. The authors who are persuasive technology researchers retrieved a total of 244 apps from App Store and Google Play, out of which 148 apps were evaluated. Two researchers independently analyzed and coded the apps and a third researcher was involved to resolve any disagreement. They coded the apps based on the persuasive strategies of the persuasive system design framework. Overall, the findings uncover that out of the 148 sustainable waste management apps evaluated, primary task support was the most employed category by 89% (n = 131) apps, followed by system credibility support implemented by 76% (n = 112) apps. The dialogue support was implemented by 71% (n = 105) apps and social support was the least utilized strategy by 34% (n = 51) apps. Specifically, *Reduction* (n = 97), *personalization* (n = 90), *real-world feel* (n = 83), *surface credibility* (n = 83), *reminder* (n = 73), and *self-monitoring* (n = 50) were the most commonly employed persuasive strategies. The findings established that there is a significant association between the number of persuasive strategies employed and the apps’ effectiveness as indicated by user ratings of the apps. How the apps are implemented differs depending on the kind of sustainable waste management activities it was developed for. Based on the findings, this paper offers design implications for personalizing sustainable waste management apps to improve their persuasiveness and effectiveness.

## Introduction

Persuasive technology is a sub-discipline of Human–Computer Interaction (HCI) that has evolved over the last 15 years. However, in recent years, the personalization of persuasive technologies has generated growing interest in the application of persuasion to technology design. Advances in smart and mobile technologies have created opportunities and shaped the way that billions of users worldwide connect and socialize with one another (Gu, 2019), learn new ways of doing things (Orji, 2017), and perform target behaviors (Istudor and Gheorghe Filip, 2014). As a result, mobile solutions such as apps and games have become attractive channels to deliver personalized and socially responsible interventions. Many of these apps and games are environmentally related and help to encourage positive individual and communal actions toward the realization of the United Nations (UN) Sustainable Development Goals (SDGs) as it concerns environmental protection and sustainability programs (such as global climate change action plans, etc.), as well as promote the health and wellbeing of the people (Nkwo et al., 2020). Specifically, these mobile sustainability apps are effective in encouraging energy conservation (Gustafsson, 2010), water preservation (Paay et al., 2013), waste management (Nkwo et al., 2018), and so on.

Sustainable waste management plays a significant role in ensuring the health and wellbeing of the people. Efforts by governments and stakeholders around the world, aimed at ensuring that citizens adopt appropriate waste disposal behaviors, have been largely ineffective (Thieme et al., 2012; Nkwo et al., 2018), hence the calls for new approaches, which can be achieved *via* the combined powers of technologies and persuasive strategies. As a result, there are increasing interests and investments in the design and adoption of technologies to change and/or reinforce sustainable waste management behaviors across the globe (Suruliraj et al., 2020b). While various studies have emphasized that sustainable waste management apps contribute to promoting clean and sustainable environmental behaviors, however, they also reported a significant amount of disuse and abandonment (Comber et al., 2013). This is because, for behavior change to occur and for continued use of the sustainability apps, developers of the apps need to employ relevant persuasive strategies (Nkwo and Orji, 2018). These strategies give the app the ability to change, reinforce, motivate, and help users to adopt sustainable environmental behaviors that are potentially beneficial to them and their communities.

Previous research has conducted a literature review on the remote causes of inappropriate waste management (Omran and Gavrilescu, 2008; Ndubuisi-Okolo et al., 2016) or the design and evaluation of persuasive apps targeting specific waste management activities (Comber et al., 2013). However, to the best of our knowledge, no study has conducted a comparative systematic evaluation of sustainable waste management apps (on Google Play or App Store) across multiple sustainable waste management activities, using the behavior change strategies from the four categories of the persuasive system design (PSD) framework (Oinas-Kukkonen and Harjumaa, 2009).

To fill this gap, we conducted a comparative systematic evaluation of 148 apps that target various waste management activities. Some of the activities include personal tracking, recycling, conference management, data collection, food waste management, do-it-yourself (DIY) projects, games, etc. The goal of this evaluation is to identify and compare the persuasive strategies employed by the apps and how they were implemented. We coded the apps based on the persuasive strategies of the PSD framework. Although there are various persuasive principles, this study chose the PSD framework for its evaluation because it is more comprehensive and yield broader findings. Moreover, they have been used successfully in recent years to deconstruct and evaluate persuasive technologies to uncover strategies employed in motivating desirable behaviors among users in various domains such as health and wellness, physical activity, and environmental sustainability such as persuasive apps for waste management.

Among others, the findings from this study show that strategies from the primary task support (PTS) category were the most implemented in the apps, followed by system credibility support (SCS) strategies, dialogue support (DS) strategies, and social support (SS) strategies in descending order. Moreover, *reduction*, *personalization*, *real-world feel* and *surface credibility*, *reminder*, and *self-monitoring* were the most commonly employed persuasive strategies. In addition, there is a substantial relationship between the number of persuasive strategies employed and the apps’ effectiveness as indicated by user ratings. Finally, we presented some design implications for tailoring such environmental sustainability apps to improve their effectiveness.

## Background and Related Literature

This section discusses literature associated with sustainable waste management. It defines the underlying principles and frameworks of persuasive designs. Also, it discusses relevant system development efforts and related literature that aimed to promote sustainable waste management activities and behaviors.

### Sustainable Waste Management

Environmental sustainability is both a huge business and a global concern in line with the global climate change campaign. This is because sustainable waste management practices play a large and important role in guaranteeing the health and wellbeing of citizens and ensures a sustainable environment (Schiopu et al., 2007; Omran and Gavrilescu, 2008; Giusti, 2009). On the other hand, improper disposal of wastes is one of the leading causes of environmental pollution (Suruliraj et al., 2020a). Incidentally, the wastes can also be reduced, reused, and recycled to produce new and useful products, if properly managed (Abdul Rahman, 2000; Sridhar et al., 2014). Studies have shown that lack of awareness and negative attitudes are some of the hindrances to efficient waste disposal, sorting, and management in most developing communities (Nkwo, 2019). As a result, governments and stakeholders around the globe had put forward several measures including awareness campaigns, legislation, and infrastructural supports, targeted at either motivating or compelling people to take on responsible waste management behaviors (Ndubuisi-Okolo et al., 2016; a et al., 2020). However, those efforts have not been effective, hence the calls for new approaches to motivate people to make behavioral and attitudinal changes. Such changes in behaviours can be realized through the combined powers of persuasion and emerging technologies. Specifically, this is when relevant persuasive strategies are implemented on user-centered technologies such as mobile phones (Nkwo, 2019).

Conventionally, persuasion involves “human communication intended to influence the autonomous judgments and actions of others” (Simons, 2011). The persuasiveness of technology is a function of its system qualities and techniques. Persuasive technologies (PTs) are interactive systems that utilize human–computer techniques or computer-mediated strategies. The strategies serve as building blocks of PTs, which are widely used in the environmental sustainability domain in general and sustainable waste management, in particular, to motivate and persuade users to change their attitudes, and support them to perform target behaviors.

### Principles and Frameworks of Persuasion Design

Over the years, researchers have propounded several persuasion principles (Fogg, 2002; Cialdini, 2006; Fogg, 2009), frameworks (Oinas-Kukkonen and Harjumaa, 2009), and the goal-setting strategy (Locke and Latham, 2002), which could be employed to design, implement, and evaluate persuasive technologies. For instance, Fogg’s functional triad and system design principles provided the original design concepts in persuasive technology development (Fogg, 2002). According to Fogg, three factors including motivation, ability, and triggers assist users to achieve their target behaviors. The main interest of Fogg’s persuasion principle is to enhance these three factors to help researchers and designers to reflect more about the target behavior that needs to be promoted/reinforced or changed and understand how to design persuasive technologies to realize the objective (Fogg, 2009). However, certain weaknesses in the principles and theories such as “inability to translate design principles into actual software requirements” saw other researchers work to improve previous design recommendations to support design and evaluation activities.

Oinas-Kukkonen and Harjumaa, in their study, developed 28 design strategies based on three stages of PS development: 1) understanding the main issue behind PS, 2) analyzing the context of PS, and 3) describing different methods to design system features (Oinas-Kukkonen and Harjumaa, 2009). The strategies are referred to as the persuasive system design (PSD) framework and are classified into four distinct categories based on the type of support that the persuasive strategies provide to users of a system and application. These include the *primary task*, *dialogue*, *system credibility*, and *social support* categories (Nkwo et al., 2018; Oinas-Kukkonen and Harjumaa, 2009).

Table 1 shows the PSD framework categories, descriptions, and persuasive strategies. Also, Table 2 shows a description of each of the strategies in the PSD framework.

**TABLE 1:** PSD framework categories, descriptions, and persuasive strategies.

Category	Description	Persuasive strategies
Primary task support	Support users in performing their intended tasks	Reduction, tunneling, tailoring, personalization, self-monitoring, simulation, rehearsal
Dialogue support	Provide feedback that moves users toward the target behavior	Praise, rewards, reminders, suggestion, similarity, liking, social role
System credibility support	Support the development of more credible systems	Trustworthiness, expertise, surface credibility, real-world feel, authority, third-party endorsements, verifiability
Social support	Motivate users through social influence	Social learning, social comparison, normative influence, social facilitation, cooperation, competition, recognition

**TABLE 2:** Description of each persuasive strategies of the PSD framework

Persuasive strategy	Description
*Reduction*	Reduces users’ effort by breaking complex behaviors into simple to help them perform the target behavior
*Tunneling*	Guide users through a process to provide opportunities to encourage them along the way
*Tailoring*	Provide information that will be more persuasive if it is tailored to the potential needs, interests, personality, usage context, or other factors related to a particular user group
*Personalization*	Offer personalized content or customized services for users
*Self-monitoring*	Allow users to track and monitor their performance, progress, or status in achieving their goals
*Simulation*	Enable users to observe the link between the cause and effect of their behaviors
*Rehearsal*	Provide means for users to rehearse their target behavior
*Praise*	Offer praise through symbols, words, images, or sounds as feedback for users to encourage their progress toward the target behavior
*Rewards*	Provide virtual rewards for users when completing their target behaviors
*Reminders*	Remind users of their target behavior to assist achieve their goals
*Suggestion*	Provide appropriate suggestions for users to achieve their target behaviors
*Similarity*	Remind users of themselves or adopt trending features in a meaningful way
*Liking*	Contain a visually attractive look and feel which meets users’ desires
*Social role*	Adopts a social role such as provide communication between users and the system’s specialists
*Trustworthiness*	Provide truthful, reasonable, and unbiased information for users
*Expertise*	Provide information showing competence, experience, and knowledge
*Surface credibility*	Contain a competent look and feel that promote system credibility based on users’ initial assessments
*Real-world feel*	Show information about people or organizations behind the content or services
*Authority*	Refer to people in the role of authority
*Third-party endorsements*	Highlight endorsements from respected and well-known sources
*Verifiability*	Provide means to investigate the accuracy of the content *via* external sources
*Social learning*	Allow users to observe other users’ performance and outcomes while they are doing the same target behavior
*Social comparison*	Allow users to compare their performances with other users
*Normative influence*	Allow users to gather with other individuals who share the same objectives to feel norms
*Social facilitation*	Enable users to discern other users who perform the target behavior
*Cooperation*	Motivate users to cooperate with other users to achieve the target behavior goal
*Competition*	Motivate users to compete with other users to achieve the target behavior goal
*Recognition*	Provide public recognition, such as ranking feature, for users

In addition, the integration and operationalization of goal-setting strategy (a non-PSD strategy) into persuasive systems has been shown to increase task performance (Locke and Latham, 2002), directs people’s attention, enhances their concentration, and lead to new approaches for performing target behaviors or tasks (van de Laar and van der Bijl, 2001).

### Persuasive Strategies Employed in Designing Persuasive Apps for Waste Management

The PSD framework has been used to design persuasive technologies to promote sustainability behaviors. For example, Thieme et al. (2012) developed BinCam, which is a two-part design, combining a social persuasive system for the collection of waste-related behaviors (Thieme et al., 2012). BinCam is intended to blend seamlessly with the everyday routine of users, with the overreaching goal of making users reflect on food wastes and recycling behaviors of young adults, a playful and shared group activity. The findings from the evaluation of the intervention showed that users found the application interactive, supportive, socially collaborative, and effective in promoting food waste management and recycling behaviors. Subsequently, the BinCam social app was later redesigned and integrated with a Facebook app to improve engagement and motivate sustainable environmental behaviors (Comber et al., 2013). The findings from that study showed an increase in both users’ awareness of, and reflection about, their waste management and their motivation to improve their waste-related skills (Thieme et al., 2012; Comber et al., 2013).

Another research carried out a user study of 153 students to discover factors that promote improper waste management behaviors among the students in a university campus in the global south. The findings from that study informed the design of a prototype waste management app, which could be used to encourage students to adopt clean and sustainable behaviors and protect the university environment *via* the provision of various personalized persuasive displays and support (Nkwo et al., 2018). The researchers employed relevant social influence strategies and personalization to tailor the design to the personal preferences and needs of the users, who were living in a closed community. Although the design was not evaluated, the results of that study demonstrated the potentials of using relevant persuasive strategies to encourage sustainable waste management behaviors among individuals and groups of people. It also showed how these strategies can be implemented on a computer and mobile technologies to help users to perform target behaviors without coercion. Subsequently, the researchers expanded their previous study to cover people living in a local community in South East Nigeria. The results of this study which were similar to the previous one were mapped to relevant persuasive strategies of the PSD framework. These strategies were used to develop socially appropriate design recommendations for building a mobile persuasive technology to promote positive waste management behaviors among communities in the global south (Nkwo, 2019).

### Persuasive Strategies Employed Based on Literature

Existing research has systematically evaluated mobile apps across several domains to establish the persuasive features they offer. For instance, in the health domain, researchers employed the strategies of the PSD framework to evaluate the effectiveness of web-based health interventions. The findings show that the intervention strategies especially the primary task support strategies were frequently implemented to encourage the adoption of healthy habits and behaviors (Kelders et al., 2012). Similarly, Orji and Moffatt (2016) conducted an empirical review of 85 papers to understand the effectiveness of persuasive technologies for health and wellness. The results of that study show that *self-monitoring*, which is one of the strategies in the primary task support category of the PSD framework, is most commonly used to operationalize persuasive health interventions (Orji and Moffatt, 2016). Based on these results, certain design recommendations were put forward to enhance the effectiveness of such health and wellness intervention. Furthermore, a systematic review of 32 papers was carried out to examine the effectiveness of social support strategies in encouraging physical activity. The results from that study show that *competition, social comparison*, and *cooperation*, which are among the strategies in the social support category of the PSD framework, were effective strategies used to motivate physical activity (Almutari and Orji, 2019). It recommended new approaches to tailor persuasive interventions to support appropriate physical activities for various categories of users. In another study, 20 research papers that presented the design and evaluation of mobile apps for promoting physical activities were systematically evaluated (Matthews et al., 2016). The results of that study showed that although some other strategies such as *reduction*, *real-world feel*, and *personalization* were incorporated in the app design, *self-monitoring*, which is one of the strategies from the primary task support category, was the prevailing strategy employed in designing the apps. In addition, previous studies had uncovered that a g*oal-setting* strategy has the potential to increase task performance (Locke and Latham, 2002), direct people’s attention, enhance their concentration, and lead to new approaches for performing target behaviors or tasks (van de Laar and van der Bijl, 2001). For instance, the results of a study that sought to suggest guidelines for designing persuasive apps to support improved breastfeeding behaviors show that such systems should allow users to set short, realistic, and measurable/trackable (self-monitoring), as well as incremental breastfeeding goals will lead to increased self-efficacy. The implication is that a relevant persuasive strategy from the PSD framework (Oinas-Kukkonen and Harjumaa, 2009) can be combined with the g*oal-setting* strategy to achieve a designed goal in a behavior-change intervention. This is important and offers great promises for designing user-centered software interventions aimed at promoting clean and healthy behaviors in the sustainability domain.

However, in the sustainable waste management sub-domain of the environmental sustainability domain, fewer recent studies have evaluated the persuasive strategies implemented in mobile apps for waste management. For instance, recent research was conducted to systematically review the persuasive strategies employed in the design of 125 sustainable waste management apps to identify the strategies from the primary task support category (alone) employed in app design (Suruliraj et al., 2020a). The results from that study showed that persuasive strategies such as *reduction*, *personalization*, *tailoring*, *self*-*monitoring*, and *rehearsal* were most commonly implemented in the apps in decreasing order. However, it also found no association between the number of persuasive strategies employed in the app’s design and its effectiveness. This is in contrast to previous studies in other domains such as physical activity (Alhasani et al., 2020), where there was some level of relationship between the number of persuasive strategies employed in the app’s design and its effectiveness. These findings draw attention to some huge gaps in research in this domain, which can be filled by a broader systematic evaluation of apps for sustainable waste management to uncover what persuasive strategies from the four categories of the PSD framework were employed in their designs.

Therefore, rather than evaluate apps to discover the persuasive strategies from the primary task support category alone, this current research article provides a comparative systematic evaluation of 148 apps across various sustainable waste management activities using the strategies from the four categories of the PSD framework (see Table 1). Specifically, we evaluated and compared the persuasive strategies from the primary task support, dialogue support, system credibility, and social support categories of the PSD framework and how they were implemented across the waste management activities such as personal tracking, recycling, conference management, data collection, food waste management, do-it-yourself (DIY) projects, and games, to uncover new insights and enrich the literature.

## Method

This study aims to conduct a systematic review of sustainable waste management apps to identify and compare persuasive strategies (from the PSD framework) employed by the apps and how they were implemented to promote appropriate waste management behaviors. Therefore, we aim to address the following research questions:

1) What persuasive strategies were employed in designing the apps for sustainable waste management?
2) How were these strategies implemented on the apps to support targeted waste management activities?
3) Is there any relationship between the number of persuasive strategies employed in the app and the apps’ effectiveness based on user ratings?

The answers to these research questions would help to inform our design recommendations for personalizing and tailoring sustainable waste management apps to improve their persuasiveness and effectiveness. The following subsections describe the apps’ selection and filtering criteria and coding process.

### Selection of Apps for Sustainable Waste Management

The app search for this study was carried out in 2020 during which we found out that most of the apps were updated last in 2019 (see Supplementary Appendix for details). We filtered our search results by selecting apps that matched with the following search terms: “waste management”, “waste disposal”, “waste recycling”, “waste tracker”, and “sustainable waste” on the App Store and Google Play. Second, we combined the search terms using “OR” and “AND” to search. The search results returned an initial list of 244 apps (App Store and Google Play).

**FIGURE 1:**
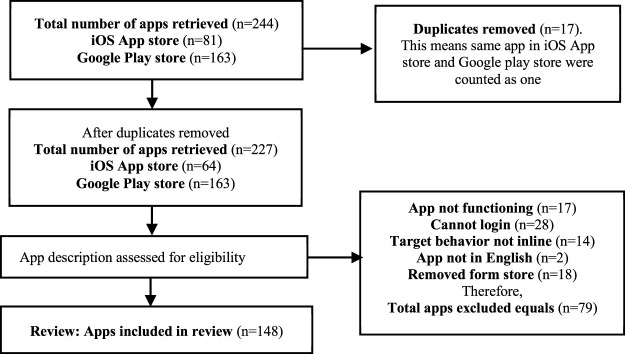
Flow diagram illustrating selection and exclusion criteria in various stages.

We employed several criteria to extract the apps that best suit the objective of the study. Primarily, we accepted only those apps that are designed to support diverse waste management activities, are free or free with in-app purchases, are in English according to the app’s description and demo, and have screenshots supplied in the description of every application. On the other hand, we excluded the apps that 1) do not support waste management activities, 2) were not described in the English language, 3) were not publicly available, 4) were outdated, and 5) cannot be logged in to explore its features and design strategies. Incidentally, the apps in this range had less than five ratings. Moreover, the researchers ensured that apps that appeared in both the App Store and Google Play were counted as one instead of two. In the end, a total of 148 apps were accepted and considered suitable for coding (see Figure 1 below). Some other information collected for each accepted app includes *application name, platform* (i.e., iPhone, Android, or both), *average rating*, *developer information*, *last update date*, and *price* (i.e., free, fee-based, and free with in-app purchases—where developers provide a free version and a paid version if users want to upgrade or unlock additional features in the app). Other information collected includes strategies implemented on the app, target outcomes, and country/region of development. We decided to choose the exclusion threshold of five ratings because it is the highest rating such apps could get from user reviews. While the apps with less than five ratings (n = 79) were excluded, apps left after exclusion were (n = 148). In other words, we selected 148 unique apps in total for coding and analysis. In addition, 85.6% of the apps were updated in 2019.

### Process of Coding Apps for Persuasive Strategies

**FIGURE 2:**
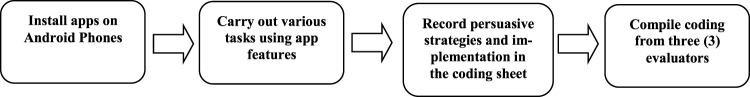
PSD categories, descriptions, and their persuasive strategies.

The purpose of coding the apps in our research is to evaluate the number and type of persuasive strategies employed in persuasive apps specifically related to sustainable waste management. Therefore, we identified the persuasive strategies (PSs) employed in designing each of the 148 sustainable waste management apps including how the strategies were implemented using the PSD framework. We chose this framework because it is more comprehensive and yields broader findings. It has been widely used in deconstructing and evaluating persuasive technologies across various domains. Two of the authors who are persuasive technology researchers installed the apps on their smartphones (Android and iOS) and used the app features to perform various tasks while taking note of the PSs integrated into them and how they were implemented, in our coding sheets. All the PSs were under the primary task support, dialogue support, system credibility support, and social support categories for coding purposes. The coding sheet was adapted from previous literature (Orji and Moffatt, 2016), validated by Nkwo et al. (2020), and modified for this research. For the features of the in-app purchase, researchers accepted the free trial to enable the examination of all persuasive strategies employed in the apps. The interrater agreement score for each strategy was computed afterward. Finally, a third expert reviewer was involved in resolving any disagreement for strategies having agreement less than 100%. Figure 2 presents the steps of coding the apps. See Supplementary Appendix for the summary of the apps evaluated and the persuasive strategies employed by the apps.

### Analysis of Data

We measured the percentage of agreement between two researchers (i.e., before the intervention of the third researcher—when needed). We also calculated interrater reliability using the percentage of agreement metric. Furthermore, we conducted descriptive statistics to obtain the average persuasive strategies employed in the design of the app. Finally, we examined the relationship between the number of persuasive strategies and the apps’ effectiveness (based on the apps’ ratings). Specifically, we performed a Pearson’s correlation analysis between the number of persuasive strategies and the app’s rating.

Computing correlation is important because it helps to explore the nature of the relationship between the two variables in question—determine which variables are most highly related to a particular outcome (Samuel and Ethelbert, 2015). Moreover, it provides the platform for regression to predict the values of the dependent variable based on the known relationship that exists between the independent variable and the dependent variable. In recent years, both the App Store and Play Store have placed a higher amount of importance on app ratings and reviews. This is because apps that have higher ratings and reviews rank high in search and have a better chance of being found and downloaded by potential users. Also, according to a recent report (Canstello, 2018), six of the most important metrics to measure apps’ success are the number of users, active users, retention, cohort analysis, and lifetime value. These metrics predominantly inform user ratings and reviews and are pointers to how effective the apps are in helping users to perform and achieve set goals.

### Agreement

The interrater reliability for the coded apps was measured using the percentage of agreement metric as explained in Albert et al. (2017). Agreement occurs when the two reviewers both indicate the presence or absence of a persuasive strategy in an app. Disagreement occurs if one reviewer indicates the presence of a strategy, and the second reviewer indicates an absence. Reliability values range between 78.6 and 100% agreement depending on the persuasive strategy. The strategies with the lowest interrater reliability (78.6%) and (82.2%) were *normative influence* and *liking*, while 26 out of the 28 strategies obtained perfect agreement scores. Generally, all intercoder reliability scores were within the acceptable range (i.e., >60%) as described by Lombard et al. (2002).

## Results

This section presents the results of the study that provide answers to the three research questions itemized in the method section. Specifically, it discusses the persuasive strategies identified in the apps and how they were implemented across target sustainable waste management activities. It also discusses the relationship between the number of strategies employed and app effectiveness.

### Information on Selected Apps

**FIGURE 3:**
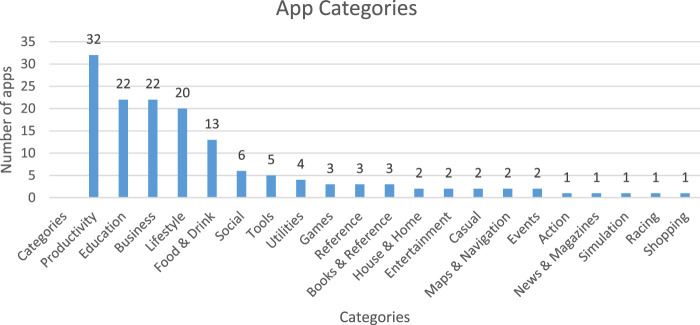
Apps in each waste management activity.

Table 3 shows the summary of the apps we downloaded and evaluated in this study. Sixty-eight percent (n = 100) of the apps were either released or updated in 2019. In addition, Figure 3 shows the number of apps in each waste management category. Detailed information about the apps can be found in the Supplementary Appendix.

**TABLE 3:** Information on accepted apps

Mobile platforms	iOS (23%), Android (77%)
User ratings	5 (5.4%), 4–4.9 (57.4%), 3–3.9 (8%), 2–2.9 (2.6%), 1–1.9 (0.6), 0 or No rating (26%)
Waste management activity category	Productivity (21.6%), Education (15%), Business (15%), Lifestyle (13.5%), Food and Drink (9%), Social (4%), Other 15 categories (22%)

### Persuasive Strategies Employed in Waste Management Apps

To answer research question 1, we downloaded 244 and evaluated 148 sustainable apps for waste management to uncover what persuasive strategies (from the PSD framework) were employed in their designs.

**FIGURE 4:**
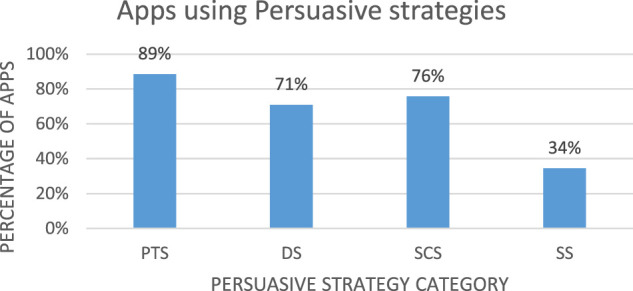
Percentage of mobile apps employing persuasive strategies.

Generally, our findings show that 27 out of 28 different persuasive strategies of the PSD framework were employed in-app designs. We did not establish the implementation of the *social role* strategy in any of the apps. The number of strategies employed in each app varied and ranges between 0 and 20. The hierarchical chart in Figure 4 shows that the *primary task support* (PTS) strategies were employed the most 89% (n = 131), followed by the *system credibility support (SCS)* 76% (n = 112), *dialogue support* (DS) 71% (n = 105), and *social support* (SS) is least 34% (n = 51). We note that most of the apps employed more than one strategy in their implementations.

Also, the results from Table 4 show that the strategies from the PTS category are the most employed in the sustainable waste management apps (sum = 327), followed by SCS (sum = 245), DS (sum = 190), and SS (sum = 75).

**TABLE 4:** PSD framework categories, persuasive strategies, and total strategies employed in apps

Category	Persuasive strategies	Total strategies in apps
PTS	Reduction (97), Tunneling (15), Tailoring (45), Personalization (90), Self-monitoring (50), Simulation (13), Rehearsal (17)	327
DS	Praise (29), Rewards (36), Reminders (73), Suggestion (33), Similarity (0), Liking (18), Social role (1)	190
SCS	Trustworthiness (16), Expertise (12), Surface Credibility (83), Real-world feel (83), Authority (11), Third-party Endorsements (8), Verifiability (32)	245
SS	Social learning (4), Social comparison (8), Normative influence (6), Social facilitation (40), Cooperation (10), Competition (3), Recognition (4)	75

**FIGURE 5:**
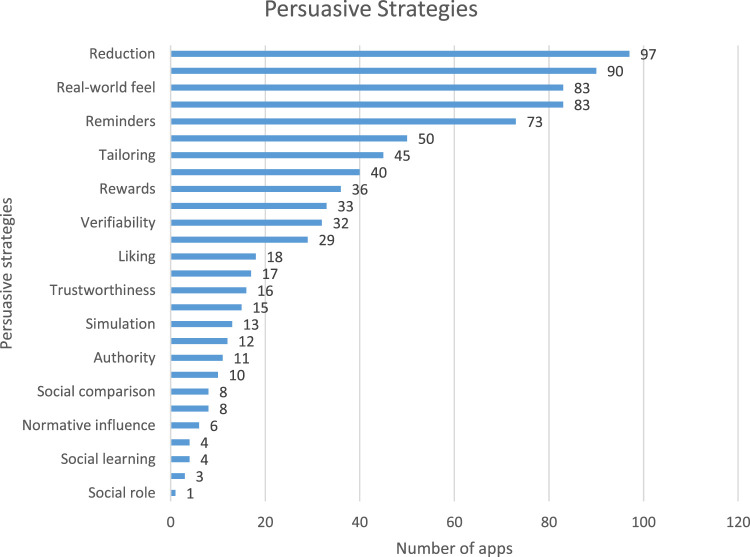
Persuasive strategies in waste management apps.

In addition, the persuasive strategies such as *reduction* (n = 97), *personalization* (n = 90), *self-monitoring* (n = 50), *real-world feel* and *surface credibility* (n = 83) each, and *reminder* (n = 73), *social facilitation* (n = 40) appear as the most frequently employed strategies in the reviewed apps. All other strategies were employed as follows: rewards (n = 36), suggestion (n = 33), verifiability (n = 32), praise (n = 29), liking (n = 18), rehearsal (n = 17), trustworthiness (n = 16), tunneling (n = 15), simulation (n = 13), expertise (n = 12), authority (n = 11), cooperation (n = 10), social comparison and third-party endorsement (n = 8 each), normative influence (n = 6), recognition and social learning (n = 4), competition (n = 3), and social role (n = 1). Please see Figure 5 for a diagrammatic description of the strategies and corresponding number of apps implementing each of them.

### Apps and Type of Waste Management Activities they were Designed for

To answer research question 2, we collected apps in 17 sub-categories based on the kind of waste management activities it was intended for (see Table 3). This was based on previous research (Suruliraj et al., 2020a). Among them, 34% (n = 51) apps were designed for regional waste disposal provided specifically to the local municipality. These apps primarily offer a garbage collection schedules calendar and waste sorting guide. Thirteen percent (n = 19) were designed to provide educational material such as articles, magazines, and news to educate people on waste management. Around 11% (n = 16) apps were focused to reduce food waste; apps in this category offer a marketplace for surplus food or track groceries in the refrigerator for expiry. Eight percent (n = 12) of the apps were used for commercial purposes and owned by private organizations. Commercial apps are used to request and manage on-demand services like dumpster rental in exchange for money. About 7% (n = 11) of the apps were designed as games; these apps will help the user to learn waste sorting by playing a sorting game and simultaneously provide facts. Some of the gaming apps offer points that can be redeemed for vouchers. In addition, 7% (n = 11) of the apps were developed for personal tracking. Personal tracking apps help users to track their daily waste management habits and show an impact chart for carbon emissions and plastics avoided. These apps can help to promote sustainable environmental behaviors. Six out of 17 sub-categories discussed previously cover 80% of the total apps evaluated in this study.

**FIGURE 6:**
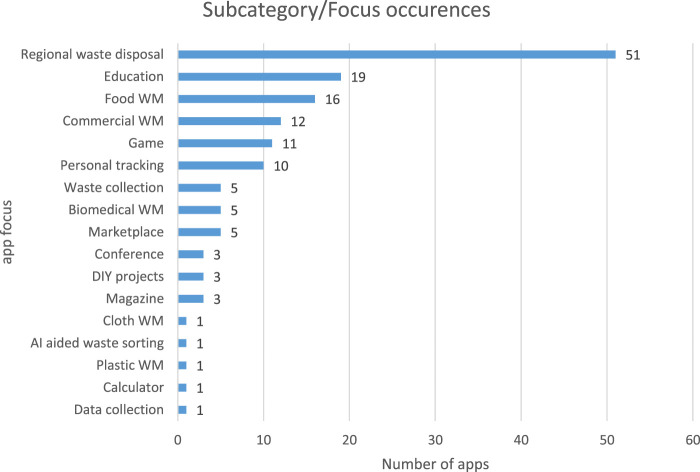
Apps and type of waste management activities targeted.

Figure 6 shows more information about other apps categorized according to their purpose and target behaviors.

**FIGURE 7:**
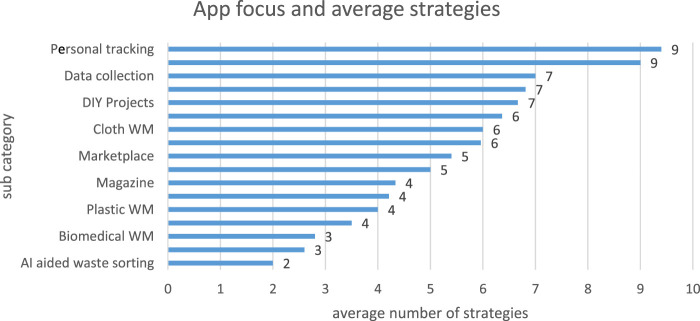
Persuasive strategies and the type of waste management activity targeted.

In addition, Figure 7 shows the persuasive strategies and types of waste management activities they were implemented for. Specifically, each of the waste management activities was operationalized with persuasive strategies as follows: Personal tracking and Conference (n = 9); Data collection, Food WM, and DIY projects (n = 7); Game, Cloth WM, and Regional waste disposal (n = 6); Marketplace and Calculator (n = 5); Magazine, Education, Plastic WM, and Commercial WM (n = 4); Biomedical WM and Waste collection (n = 3) and AI-aided waste sorting (n = 2).

### Persuasive Strategies Implementation in the Apps

Generally, persuasive strategies are used to motivate and influence users to reach their personal and group goals through user engagement and collaboration. However, in this section, we present the distinct implementations of the strategies of the PSD framework, which are frequently employed in sustainable waste management apps.

#### Primary Task Support Strategies

The primary task support (PTS) strategies support individuals and groups to perform their primary tasks (Oinas-Kukkonen and Harjumaa, 2009). We found that 89% (n = 131) of the sustainable waste management apps implemented the strategies from the *primary task support* (PTS) category of the PSD framework (see Figure 4). The commonly implemented strategies in the PTS category are *reduction*, *personalization*, and *self-monitoring* among others (see Figure 5). Specifically, *reduction* strategies, which “reduce complex tasks into simpler ones so that system users can perform target behaviors easily” (Nkwo and Orji, 2018), were implemented in 97 apps as suggestive search (auto-populate listing) to reduce efforts in searching for relevant information. Other apps implemented it as a calendar view with color-coding to reduce time spent in searching for a garbage collection schedule, QR code/Bar code scan, and log in using third-party apps like Facebook and Google. *Personalization* strategies offer personalized content, functionalities, and services to users (Oinas-Kukkonen and Harjumaa, 2009), and were implemented in 90 apps as personalized language settings. These allowed users to choose the preferred languages with ease. Other apps implemented it through personalized notification times, email reminders, save location, user profiles, and personalized setting of user preferences and payment options. *Self-monitoring* strategies, which “allow people to keep track of their performances, offering information on both past and current behaviors” (Orji, 2017), were implemented in 50 apps as exclusive app screens to review trends of individual data related to history, statistics, environmental impact, and amount of CO_2_ wastes released. The gaming apps implemented it *via* a real-time display of the player progress points earned and levels completed per game session.

#### System Credibility Support

The system credibility support (SCS) strategies describe how to design a system to be more credible and persuasive (Oinas-Kukkonen and Harjumaa, 2009). Seventy-six percent (n = 112) of the sustainable waste management apps implemented the strategies from the system credibility support (SCS) category of the PSD framework (see Figure 4). The commonly implemented strategies in the SCS category are *real-world feel* and *surface credibility* among others (see Figure 5). While *real-world feel* strategies provide information about the owners of the system, *surface credibility* strategies offer a competent look and feel for users (Nkwo and Orji, 2018). The real-world feel and surface credibility strategies were both implemented in 83 apps each through “about us/contact us pages”, “terms of service”, “privacy policy”, version information with date, Frequently Asked Questions (FAQ) section, list of services offered, website information, and map view.

#### Dialogue Support Strategies

The dialogue support (DS) strategies offer some measure of system feedback to system users (Alqahtani et al., 2019). We uncovered that 71% (n = 105) of the sustainable waste management apps implemented the strategies from the DS category of the PSD framework (see Figure 4). The commonly implemented strategy in the DS category is *reminder* among others (see Figure 5). *Reminder* strategies allow a system to remind the user to perform target behaviors (Nkwo and Orji, 2018). They are implemented in 73 apps as push notifications to remind users about disposing of garbage, food item expiration alerts, news, and suggestions, etc. Other apps implemented it alongside self-monitoring strategies to remind users to track their data and status, and/or to perform certain waste management activities such as waste sorting, garbage collection, evacuation of waste bins *via* email reminders, text messages, pop-ups, and sounds.

#### Social Support

The social support (SS) strategies describe how to design a system to support users to perform target behaviors by leveraging social influence (Nkwo et al., 2020). We uncovered that 34% (n = 51) of the sustainable waste management apps implemented the strategies from the SS category of the PSD framework (see Figure 4). The frequently implemented strategy in this category is *social facilitation* among others (see Figure 5). *Social facilitation* strategy allows a system to offer a means to discern other individuals who are performing the target behavior (Nkwo and Orji, 2018). This strategy is implemented in 40 apps in the form of a community forum of users and regional waste managers. Connected users could see each other’s activities, concerns, and suggestions or planned waste management activities. This will set the stage for users to exchange views or cooperate to tackle certain waste management issues and concerns *via* shared social communities such as a Facebook group for the app.

### Persuasive Strategies and App Effectiveness

To answer research question 3, we ran the Pearson correlation coefficient (*r*) to determine whether any relationship exists between the number of persuasive strategies implemented in the apps and the apps’ perceived effectiveness (based on average ratings). The computation was performed for all the apps combined. The results revealed that *r* (146) = 0.21, *p =* 0.012. The result means that overall, there is a significant correlation between the number of persuasive strategies employed and app effectiveness. This relationship that exists confirms the perceived effectiveness of the apps to promote sustainable waste management behaviors, from the user’s point of view. Nevertheless, it is possible but unlikely that the correlation would change in this study’s current state when different exclusion criteria and values are picked. This is because the exclusion criteria applied in filtering the apps with less than five ratings are fixed. In specific terms, we excluded the apps that did not support waste management activities, were not described in the English language, were not publicly available and those that cannot be logged in to explore its features and design strategies.

**FIGURE 8:**
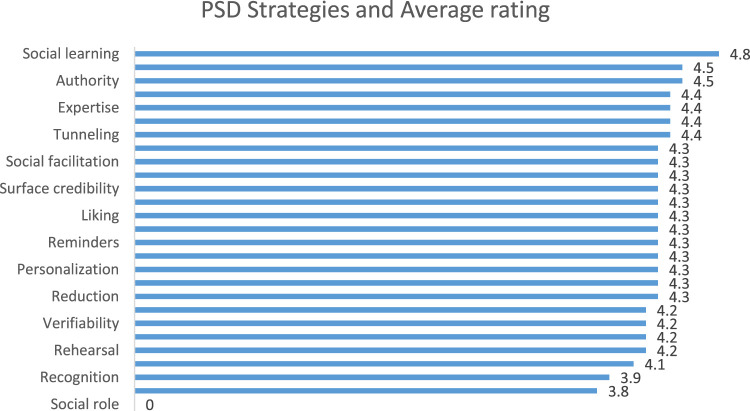
Relationship between persuasive strategies and app ratings.

Furthermore, Figure 8 shows that apps using the “*social learning*” strategy have the highest average rating of 4.8. All other strategies have their ratings as follows: “social comparison” and “authority” (4.5 each), “third-party endorsement”, “expertise”, “simulation” and “tunneling” (4.4 each), “cooperation”, “social facilitation”, “real-world feel”, “surface credibility”, “trustworthiness”, “liking”, “suggestion”, “reminders”, “self-monitoring”, “personalization”, “tailoring” and “reduction” (4.3 each), “normative influence”, “verifiability”, “rewards” and “rehearsal” (4.2 each), “praise” (4.1), except “r*ecognition*” and “*similarity*” strategies with 3.9 and 3.8, respectively. Only one app employed the “s*ocial role*” strategy, but the app did not have any rating and was excluded. The average rating is a measure of what a given customer base or population, on average rates a certain product or service. It is computed using the following equation given the total number of ratings at each level.

AR = (1*a) + (2*b) + (3*c) + (4*d) + (5*e)/5

Where AR is the average rating, a is the number of 1-star ratings, b is the number of 2-star ratings, c is the number of 3-star ratings, d is the number of 4-star ratings, and e is the number of 5-star ratings.

## Discussion

In this section, we discuss the results of our study and offer some design recommendations for sustainable waste management apps based on our results and conceptual analysis as well as other relevant research.

### Persuasive Strategies and Implementation

The goal of this research is to identify distinct persuasive strategies integrated into the apps developed to promote sustainable waste management behaviors and group the strategies based on the type of waste management issues or activities that the app targets or focused on. Furthermore, the study aims to uncover how the persuasive strategies were implemented in sustainable waste management apps to achieve their intended purposes, and also to examine the relationship between the persuasive strategies employed and apps’ effectiveness.

First, this subsection provides answers to research question 1. It discusses the relevant persuasive strategies employed in designing the apps. Overall, the sustainable waste management apps reviewed in this paper employed 27 persuasive strategies. The implementation ranges from minimum (0) to maximum (20) per app.

### Primary Task Support Strategies

Predictably, we uncovered that the persuasive strategies from the primary task support (PTS) category of the PSD framework were the most employed in the apps 89% (n = 131). Among the strategies in this category, we discuss the implementation of three different strategies: *reduction, personalization*, and *self-monitoring.* We opted to discuss these three strategies because they are the most commonly employed strategies in the evaluated mobile apps. This is in agreement with a previous study (Suruliraj et al., 2020a), which shows that the primary task support strategies such as *reduction*, *personalization*, and *self-monitoring* among others are considered the main features of many sustainability interventions.

Reduction strategies emerged as the most implemented strategy (n = 97) and help users reduce efforts and simplify complex tasks into simpler ones so that users can be able to perform target behaviors with ease. The implementation of this strategy enables users to be able to search for relevant information such as the nearest public waste bucket, garbage collection schedules, etc. *via* a calendar view with color-coding. This reduces search time. System interventions that provide easier avenues to carry out target behaviors would motivate users to engage with and continue with the behaviors. These results demonstrate that the intervention strategies from the primary task support could be effective in helping individuals and groups to carry out their basic tasks or activities with ease. We refer to this attribute as “user-friendly routines”**.** This finding is in agreement with previous studies (Oinas-Kukkonen and Harjumaa, 2009).

Personalization strategies emerged as the second most implemented persuasive strategy (n = 90) in sustainable waste management apps. Price et al. (2016) opine that allowing users to change colors, set backgrounds, and make other personalized settings on an app would improve its usability (Price et al., 2016). The ability to regulate the system intervention delivered *via* sustainable waste management apps to suit the user’s needs and characteristics will make the system more effective. Moreover, studies have shown that personalized persuasive technologies are more effective at motivating users to perform target behaviors than the one-size-fits-all method of design (Moses et al., 2018). This is also true for sustainable waste management interventions in particular according to a recent study (Suruliraj et al., 2020a). So, it is unsurprising to see that sustainable waste management apps integrated some form of personalization because potential users may have unique needs and requirements based on factors such as literacy level, etc. This strategy will improve the user-friendliness of the app. We refer this attribute to as “adaptive design”. This will allow users to customize certain functionalities of the app to improve its usefulness.

Self-monitoring is the third most employed strategy of the primary task support. It helps users of sustainable waste management apps to keep track and effectively manage their performances and goals (Matthews et al., 2016; Orji et al., 2018). Users can track their feeling, thoughts, and behaviors, which in turn increases self-awareness and motivate sustainable behavior outcomes. Most of the apps allowed for manual data entries and automatic display of information and statuses in the English language. Manual entries may be difficult and time consuming, and the display of user statuses in non-indigenous languages may not work for people with low literacy levels as they will not be able to read and write in English. The results demonstrate that many individuals or groups will be more motivated to embark on a task if they are provided with the means to keep track of their performance or status. We refer to this attribute as “performance tracking”. Performance tracking is supported by intervention strategies such as self-monitoring, recognition, praise, and goal-setting. This finding is in line with previous studies (Orji et al., 2012, van de Laar and van der Bijl, 2001).

### System Credibility Support Strategies

The persuasive strategies from the system credibility support (SCS) category of the PSD framework were the next most employed strategies in the apps 76% (n = 112). The credibility strategies such as *real-world feel* and *surface credibility* among others were implemented in sustainable waste management apps.

Real-world feel along with surface credibility emerged as the most implemented credibility strategy in the apps, and it provides information about people or organizations behind the app’s content (Nkwo and Orji, 2018). It is offered in 83 apps. We argue that this strategy is essential in sustainability interventions. Like other interventions, apps for sustainable waste management should provide relevant and home-grown instructions, guidelines, and tips that are environmentally friendly and socially appropriate to users in a particular community. Anyone can design apps and publish them on the apps store, but technical and development skills are not sufficient for building apps that will effectively promote sustainable behaviors.

Surface credibility strategy is also offered in 83 apps. It ensures that the app offers a professional look and feel, to make a positive impression to users assessing the apps’ contents and services (Nkwo and Orji, 2018). Considering that users will be supplying their sensitive information such as residential addresses, they need to be assured that their data are in credible hands. Full disclosure of owners’ information and competent look and feel make an app credible (Oinas-Kukkonen and Harjumaa, 2009). Hence, providing opportunities for users to contact the app owners to make inquiries or ask questions and receive feedback from the apps, as well as ensuring a cleaner interface will improve the credit rating of an app.

### Dialogue System Strategies

The persuasive strategies from the dialogue support (DS) category of the PSD framework were the third most employed strategy in the apps (71%, n = 105). The dialogue support strategy such as *reminders* among others was implemented in apps.

A reminder strategy is designed to remind users and improve their observance of desired behaviors. It reminds individuals about waste collection dates and locations, disposal of garbage, tracks their personal information, and to perform some helpful sustainable waste management activities such as sorting. However, studies have shown that multiple and unsolicited reminders could annoy a user and lead to de-motivation and eventual disengagement (Bakker et al., 2016). There is therefore the need to take special cautions in implementing reminders in an app to avoid annoying users. One of the ways to achieve this result in an app is to tailor reminders to each individual or group. According to Alqahtani et al. (2019), tailoring reminders is significant because individuals and groups can be allowed to customize the frequency at which reminders are sent to them (how often), but also the type of reminder (pop-up boxes, text message, sounds, etc.) and when it should be sent (time). The results show that the strategies from the dialogue support could be useful in providing some degree of system feedback to its users, potentially through automated text messages, and pictorial or verbal information. We refer to this attribute as “automated notification management”. This finding is in line with previous studies (Orji et al., 2012).

### Social Support Strategies

The persuasive strategies from the social support (SS) category of the PSD framework were the fourth most employed strategies in the apps 34% (n = 51). Among other strategies in this category, *social facilitation* was the most implemented in the apps (see Figure 5).

Social facilitation is designed to provide a way to discern other individuals who are performing the target behaviors (Nkwo and Orji, 2018). It was implemented in 40 apps. Systems that offer opportunities for users to share their thoughts and concerns with similar others and build synergy with them will help to improve engagement. Users can share app-supplied information with other users *via* text, social media, email, or other means, depending on the device options. Therefore, developers of apps for a sustainable environment should focus on incorporating social facilitation features that allow users to recognize other users performing the same behaviors. This way the app will be more persuasive. Leveraging social influence strategies such as social facilitation could help shape users’ behaviors. We refer to this attribute as “social support”. This finding is in line with previous studies (Oinas-Kukkonen and Harjumaa, 2009).

### Persuasive Strategies Implemented and Type of Waste Management Activities

Secondly, this subsection provides answers to research question 2. It discusses the type of sustainable waste management activities that the persuasive apps were designed for and how relevant persuasive strategies were implemented to support those activities. As can be seen from Figures 6 and 7, nearly all the sustainable waste management apps that we reviewed in this study targeted a mixture of waste management issues or activities. This makes it difficult to determine which persuasive strategies are more effective for a definite waste management activity. However, *reduction*, *personalization*, *self-monitoring* (primary task support), and *reminder (dialogue support)*, *real-world feel* and *surface credibility (system credibility support)*, and *social facilitation (social support)* are the most employed persuasive strategies in various sustainable waste management activities.

In general, the apps mostly targeted the following sustainable waste management issues or activities: personal tracking, conference management, data collection, food waste management, do-it-yourself (DIY) projects, games, and so on (see Figure 7). Specifically, apps for personal tracking and conference management employed the most number of strategies averaging nine strategies per app, followed by apps for data collection, food waste management, and DIY projects each with an average of seven strategies per app. Mobile apps that were designed as a game (waste sorting and recycling), cloth waste management, and regional waste disposal each implemented an average of six strategies. The marketplace and calculator apps employed an average of five strategies; apps focusing on the magazine, education, plastic waste management, and commercial waste management employed an average of four categories each. Mobile apps in the biomedical waste management and waste collection subcategories are second to the last, implementing an average of three strategies and artificial intelligence (AI)–aided waste management app implemented the least number of strategies; 2. For details, see Figure 7.

### Persuasive Strategies Implemented and App Effectiveness

Thirdly, this subsection provides answers to research question 3. Specifically, the effectiveness of the apps was measured based on the app’s rating. Interestingly, we established a significant relationship between the number of persuasive strategies and apps effectiveness as indicated by user ratings. This is particularly an interesting result considering the recent discussion and open research question on whether persuasive systems employing multiple persuasive strategies are more effective than those employing a single strategy (Orji, 2017). Our result implies that employing multiple strategies will increase apps’ effectiveness in the area of waste management. This is not so with results from previous research in the health domain, which shows that employing one strategy can be effective (Alqahtani et al., 2019).

A possible explanation for the difference can be found in the differences inherent in the domains of investigation. This study targets sustainable waste management while the previous studies focused on health. For the previous study, it may seem that many people are conscious of their health since it has a personal and direct impact on their wellbeing—hence, they could easily be persuaded to adopt a healthy behavior. However, this is not the same with the sustainability domain (especially sustainable waste management), which has more of an indirect and most time community-level effect. It may take some extra effort to motivate people to adopt sustainable waste management behavior since it is difficult to show the cause-and-effect of each individual’s behaviors and their contributions to the global, national, and community sustainable development goals (SDGs). Hence, designers and other stakeholders must focus on selecting the appropriate combination of persuasive strategies for an app, having both the target users and target activities in mind.

### Comparative Evaluation of Dominant Persuasive Strategies

Table 3 describes the leading persuasive strategies employed in the apps. In a fast-paced world where ease of access and exactness are needed, *reduction* and *personalization* are certainly vital to tailor sustainability apps to individual users. It is therefore not surprising that reduction and personalization are the most dominant and most implemented in sustainable waste management apps. Users tend to be critical and may abandon apps if it is not user-friendly and does not support personalized access. While *reminders* and *suggestions* are important for notifying, reminding, and providing feedback to users to perform a target behavior, *praise* and *reward* are essential for providing positive reinforcements using virtual praise and/or rewards (e.g., texts or badges or sounds) or real rewards (e.g., coupons). These are important for the continued performance of target behaviors. *Self-monitoring* is also dominant in sustainable waste management apps since technological advancements have made it possible to automatically track personal and performance data over time, public trash can, etc., in real time through various sensors on smartphones, wearable devices, and public facilities. This will help users and managers to visualize their daily contributions to a clean and sustainable environment, and help them become more responsible and conscientious citizens of the society. It is also possible to monitor food wastes and carbon monoxide emission levels in industrial settings using tracked information. This explains why self-monitoring is among the top in the domain of environmental sustainability. *Surface credibility* and *real-world feel* are important for integrity, emotion, and positive feelings, due to the sensitive nature of these apps. Users tend to be skeptical and critical of apps in these areas and that makes it essential that the apps must be professional-looking, responsive, and with a visually appealing interface to be adopted. Any app that lacks these attributes may be deemed incredible. Hence, surface credibility is one of the popular strategies in the sustainability domain. Relevant social influence strategies such as *normative influence*, *social facilitation*, and *social role* are significant and useful in motivating individuals and groups of users to perform desirable waste management behaviors through positive peer pressure, evidence-based information displays, etc.

### Design Implication

In this section and based on our findings, we offer design suggestions for tailoring sustainable waste management apps to improve their persuasiveness and effectiveness. In addition, we carefully integrated into our design recommendations some findings from relevant research (such as goal setting—a non-persuasive system design strategy), which will potentially strengthen some of the persuasive features of the app and hence improve its effectiveness (see Table 5 **).**

1) User-friendly Routines: Accessibility and the ease of use of the various features of the app may have a significant influence on the user’s behaviors toward task performance. Therefore, the designer should employ the *reduction* strategy in apps that target sustainable waste management to help users to perform their primary tasks with less difficulty and when required. Providing essential and easily accessible features such as shortcut menus, single-click or one-button press commands to commonly requested waste management issues such as collection and disposal locations and times, waste sorting, etc., would reduce complex behaviors for busy people and encourage them to imbibe appropriate waste management lifestyles even on the go behaviors (Nkwo and Orji, 2018). For example, the app may be customized to list the locations of nearby public waste bins in a community. This feature could be configured (using Google Maps) to automatically detect the user’s current location and suggest the closest waste drop-off location, thereby helping users to preplan their routes to work/business and dispose of their wastes at the appropriate places. Moreover, because of the low literacy rate in certain communities, especially in the Global South, technical knowledge or extensive smartphone usage skills cannot be assumed for every user of such mobile apps. Therefore, designers should simplify the process by presenting the most frequently accessed features and easy-to-use features to the potential users of the apps, all advanced features that can be accessed by experienced users may require more steps to access them. This will help reduce the amount of effort and time that users spend trying to figure out how to use the mobile app to perform an activity and focus on the intended waste management activity.
2) Adaptive Features: Offering personalized content and features which will allow users to adapt some app functionalities to suit their individual preferences will go a long way to motivate the performance of target behaviors and may increase the apps’ effectiveness (Nkwo and Orji, 2018). Adjusting app features such as the font size, type, and color of texts, background, layout, type of wastes you want to dispose of, waste management activities that users want to engage in, etc., based on user’s data, would improve the usefulness of the sustainable waste management interventions. Moreover, given that many sustainable waste management apps target more than one waste management issue or activity, it becomes imperative that designers adapt the apps based on the type of waste management issues or activities that each experience. In addition, individuals who may be engaged in similar or same waste management activities may have unique needs that require personalized attention, hence emphasizing the need to personalize sustainable waste management apps to each need. Similar to system-controlled adaptation, designers can enable user-controlled adaption (customizations). This will allow users to adapt the features and functionalities of the applications to suit their needs. Research shows that both approaches to adaptation share common strengths of increasing users’ perception of a system’s relevance, usefulness, interactivity, ease of use, credibility, and trust, and also increases users’ self-efficacy (Orji et al., 2017). However, there are notable differences between system- and user-controlled adaptation. User-controlled adaptation gives users a sense of *freedom*, *control*, and *personal touch* over the system, which in turn increases their commitment and hence systems effectiveness. System-controlled adaption reduces the app complexity (Orji et al., 2017). Therefore, we recommend that app designers can employ both, providing some adaptable features that users can control themselves, including background color, font, allowing app features to be enabled or disabled, and removing unnecessary categories that do not apply to their waste management needs.
3) Automated Intelligent Notification Management: Providing intelligent reminders to notify the user to perform their target behaviors or keep track of certain waste management activities would help to motivate sustainable waste management behaviors and increase the apps’ effectiveness (Oinas-Kukkonen and Harjumaa, 2009). For example, the designer can implement a feedback mechanism to remind the user to dispose of the right kind of waste at the right time, notify a user about a food’s expiry, or exciting waste-for-cash offers in nearby waste collection locations. For mobile apps that support personal tracking of waste disposal habits, persuasive reminders that motivate/reinforce positive benefits and reward compliance can motivate users to continue with desirable waste management behaviors. This aligns with research that shows that positive reinforcement and gain-framed appeal are possible intervention strategies for strengthening people’s behaviors (Orji, 2017). Positive reinforcement (Wilson, 2003) can be achieved by rewarding every sustainable waste management act (“*praise*” and “*rewards*” strategies) using virtual praise and/or rewards (e.g., texts or badges or sounds) or real rewards (e.g., coupons). On the other hand, gain-framed appeal refers to notifications that focus on the benefits of adhering to or performing a target (Wansink and Pope, 2015) (e.g., waste disposal, waste sorting) and can be facilitated using the *suggested* strategy. For example, gain-framed messages like “*By sorting your waste appropriately, you’ll get a chance to earn some cash.*” can be sent at specified times to people motivated. Multiple and unsolicited reminders could annoy a user and lead to de-motivation and eventual disengagement (Bakker et al., 2016). To avoid this scenario, designers should tailor reminders to each individual or group. The act of tailoring reminders would allow app users to customize the frequency at which reminders are sent to them (how often), but also the type of reminder (pop-up boxes, text message, sounds, etc.) and when it should be sent (time).
4) Performance Tracking: The designers should employ a self-monitoring strategy in apps that target sustainable waste management activities to track their data and performance over time. Allowing individuals to track their performance and visualize their data (performance statuses) in attractive formats would offer the opportunity for self-awareness and evaluation, and help them to become more responsible in managing their wastes. For example, if a user is convinced that reducing his daily level of carbon-dioxide emission in the locality is beneficial, there is a possibility that he will continue to perform target behaviors. Also, performance tracking can be achieved *via* the design of mobile apps that tracks and updates the display of user contribution to a clean and sustainable environment by cutting down plastic use, reselling old electronics, up-cycling old items, etc. An impact chart with categories of waste will potentially help the user to visualize their progress which may engender self-efficacy. Some behavior data cannot be automatically monitored without users’ involvement due to technology limitations. Therefore, for such behaviors, designers should provide some forms of praise and/or reward to users for tracking their behaviors each day. Performance tracking techniques have been used to support motivated people, especially those who are experienced in the potentialities of such interventions, to achieve target behaviors. However, according to previous studies, inexperienced users will likely be more demotivated in the process of using performance tracking interventions (Rapp and Cena, 2016). This is not unconnected to cumbersome tasks associated with personal information collection, nonfigurative visualizations, and the use of technology (Rapp and Cena, 2016). This will even be more evident in local communities in the Global South as such behavior-change apps would be deployed among potentially low-literate users who may be more disinclined to new technology adoption. Therefore, there is a need to employ complementary strategies that will take away the cumbersome tasks and expectations from users of the app. In addition, taking the job away from users and automating the collection of personal data and display of relevant information to users in visually attractive and descriptive formats would motivate the usage of such apps among less literate users. The *reduction*, *similarity*, and *liking* strategies could be employed to achieve this purpose. They should be integrated to reduce the number of efforts needed to perform target behaviors, and remind users about themselves and desired target behaviors in a visually attractive manner. Other corresponding persuasive strategies such as *reminders* and *suggestions* should also be operationalized on such apps to remind and help users to track and record their data. Self-efficacy can be enhanced through self-commitment by setting short-term goals (van de Laar and van der Bijl, 2001). The integration of the “*goal-setting*” strategy will motivate task performance, channel people’s attention and focus on desired behaviors, enhance their awareness, and lead to new approaches for succeeding in the task (Locke and Latham, 2002, van de Laar and van der Bijl, 2001). The goal should be incremental (Orji, 2017); in other words, as an individual’s confidence grows, the set goal could be reviewed upwards. Hence, sustainability interventions such as waste management games/apps should allow users to set short, realistic, and measurable (*self-monitoring*), as well as incremental sustainable waste management goals. This will lead to increased self-efficacy.
5) Credible and Responsive Design: The apps should be designed to provide potential users with relevant and home-grown sustainable waste management instructions, guidelines, and tips that are socially appropriate to a particular community. Anyone can design apps and publish them on the apps store, but technical and development skills are not sufficient for building apps that will effectively promote sustainable behaviors. Thus, the app should offer waste management information that is endorsed by expert third parties. The users should also be able to verify the reliability of the information presented on the app. This will increase app reliability and encourage users to engage with the app. Moreover, surface credibility ensures that the app offers a professional look and feel, to make a positive impression to users assessing the apps’ contents and services (Nkwo and Orji, 2018). Considering that users will be supplying their sensitive information such as residential addresses, they need to be assured that their data are in credible hands. Full disclosure of owners’ information and competent look and feel make an app credible (Oinas-Kukkonen and Harjumaa, 2009). Hence, providing opportunities for users to contact the app owners to make inquiries or ask questions and receive feedback from the apps, as well as ensuring a cleaner interface will improve the credit rating of an app.
6) Social Support Design: Employing strategies that leverage social influence to design apps for sustainable waste management will provide users the opportunity to share their experiences and support one another to perform target behaviors. A user can be able to discern others who are engaged in similar waste management activities and would be motivated to share her experiences and concerns with them (social facilitation). They can also share the app contents on other media (SMS, WhatsApp, Facebook, etc.), which helps to spread the word and will help to bring like-minded people together. Using the “*normative influence*” strategy, positive peer pressure can be applied to enhance the possibility that an individual will adopt positive waste management behaviors. For instance, education mobile apps that offer evidence-based sustainable waste management information and community resources (including inspiring photos/videos, success stories, testimonials, etc.) for educating and influencing changes in beliefs, narratives, or attitudes can be disseminated to target groups. This could be done through discussion forums (peer-to-peer, stage-matched, or moderated peer-to-peer forums), online mutual-help support communities, asynchronous bulletin boards, and virtual chat rooms. In addition, the “social role” strategy through the ask-a-waste-manager service can help to support individuals toward sustainable waste management.

**TABLE 5:** Practical recommendations for design and associated persuasive strategies

Recommendations for design	Persuasive strategies
User-friendly routines	Reduction
Adaptive features	Personalization, tailoring
Automated notification management	Reminder, praise, reward, suggestion
Performance tracking	Self-monitoring, goal-setting, recognition, praise, reminder, suggestion
Credibility and responsiveness	Real-world feel, surface credibility
Social support design	Social facilitation, normative influence, social role

## Limitations

This study has several limitations. One of them is that we reviewed only apps that were provided in the English language. Since there are apps that are in other languages, the results may not be generalizable. Second, due to the dynamic nature of the Google Play and iOS App stores, the composition and features of the apps we reviewed could be altered by the time this paper is published. In addition, user ratings may not be enough to ascertain the effectiveness of apps. This is because many other factors can influence the effectiveness of apps. However, user rating was the singular, closest evaluation we had to measure effectiveness.

## Conclusion

Our society has become a platformized one. Mobile technology, which is one of the major features of our society, is a major influencer and could be employed to promote sustainable behavior change. This article provides a systematic evaluation of mobile apps for sustainable waste management to deconstruct and compare the persuasive strategies employed and their implementations.

The results from this study show that strategies from the primary task support, followed by system credibility support, dialogue support, and social support categories, were implemented at various levels. Specific persuasive strategies such as *reduction*, *personalization*, *real-world feel* and *surface credibility*, *reminder*, and *self-monitoring* were regularly used to design the apps for sustainable waste management such that it could motivate users to perform target behaviors. Moreover, it discovered that there is a relationship between the number of persuasive strategies employed and the effectiveness of the apps. Lastly, based on the results, we presented design implications for tailoring such persuasive apps for sustainable waste management to improve their effectiveness. In future research, experimental work will be required to show the guideline’s applicability in the actual design and usage situation of persuasive technologies for sustainable waste management in particular and environmental sustainability in general. Future studies will also examine which persuasive strategies are most important to users in achieving sustainable waste management goals.

## Data Availability

The original contributions presented in the study are included in the article/Supplementary Material, further inquiries can be directed to the corresponding authors.
