# Supplementary material for: Persuasive Apps for Sustainable Waste Management: A Comparative Systematic Evaluation of Behavior Change Strategies and State-of-the-Art
Source: Front Artif Intell. 2021 Dec 9;4:748454. doi: 10.3389/frai.2021.748454 (PMC8696078; doi:10.3389/frai.2021.748454)
Supplement: Supplementary file 1 [file Table1.pdf]

Appendix

S No	Application name	Platform	Last update	Category	Sub-category/Focus	No.of. Ratings	Average Rating	Persuasive Strategies	Total PSD Strategies
1	1 Million Women	iOS	Nov 13, 2019	Lifestyle	Personal tracking	10	4.8	Reduction, Tailoring, Personalization, Praise, Reminders, Expertise, Real-world feel	7
2	Bay Disposal	Android	November 25, 2016	Business	Waste collection	0	0	Expertise, Real-world feel, Authority	3
3	Bee2a Waste recycling technology	Android	November 23, 2017	Business	Regional waste disposal	16	4.5	Surface credibility, Real-world feel	2
4	Benton County Waste Management	Android	August 1, 2019	Productivity	Regional waste disposal	0	0	Praise	1
5	Best Before - Food Tracker	Android	November 2, 2017	Food & Drink	Food WM	188	4.1	Expertise, Surface credibility, Third-party endorsements	3
6	Big Dumpster	Android	January 23, 2019	Business	Commercial WM	29	4.2	Reduction, Personalization, Surface credibility, Real-world feel, Authority, Normative influence, Social facilitation	8
7	BIN IT	Android	March 9, 2019	Education	Regional waste disposal	40	4.1	Reduction, Tunneling, Personalization, Rewards, Trustworthiness, Surface credibility, Real-world feel	8
8	Bin The Trash: Recycling Game	Android	June 3, 2018	Casual	Game	15	4.7	Reduction, Tailoring, Personalization, Reminders, Surface credibility, Real-world feel	6
9	Biomedical Waste Management Learning App	Android	March 27, 2018	Education	Biomedical WM	56	4.7		0
10	Bukkawaste	iOS	Sep 24, 2019	Productivity	Waste collection	7	4.4	Personalization	1
11	Calgary Garbage Day	Android	August 1, 2019	Productivity	Regional waste disposal	3250	4.7	Expertise, Surface credibility	2
12	City of Bunbury My 3 Bins	iOS		Utilities	Regional waste disposal	0	0	Rewards, Reminders, Liking, Real-world feel, Social facilitation	5
13	City of Hamilton	Android	July 18, 2019	Social	Regional waste disposal	12	4.1	Personalization, Rewards, Authority	3

14	City of Peterborough Waste	Android	August 1, 2019	Productivity	Regional waste disposal	46	4.5	Reduction, Tailoring, Personalization, Rehearsal, Praise, Rewards, Reminders, Surface credibility, Real-world feel, Verifiability	10
15	City of St Albert: BeWasteWise	Android	August 1, 2019	Productivity	Regional waste disposal	108	4.8	Reduction, Tailoring, Personalization, Reminders, Surface credibility, Real-world feel, Verifiability	7
16	Clean India - Recycle Waste	Android	June 15, 2015	Education	Game	44	4.5	Simulation, Praise, Suggestion, Liking, Surface credibility, Real-world feel	6
17	Coffs Waste Conference	Android	April 18, 2019	Events	Conference	0	0	Reduction, Tailoring, Personalization, Rehearsal, Praise, Rewards, Reminders, Surface credibility, Real-world feel, Verifiability	10
18	CoGo - Connecting Good	iOS	Nov 15, 2019	Lifestyle	Personal tracking	36	3.6	Reduction, Tunneling, Personalization, Trustworthiness, Surface credibility, Real-world feel, Social facilitation	8
19	Colchester Waste Management	Android	August 1, 2019	Productivity	Regional waste disposal	142	4.7	Reduction, Tailoring, Personalization, Reminders, Surface credibility, Real-world feel, Verifiability	7
20	Commurban	Android	September 30, 2019	Social	DIY projects	5	5	Reduction, Tailoring, Personalization, Reminders, Surface credibility, Real-world feel, Authority, Verifiability	8
21	Covanta Games	iOS	Jun 13, 2018	Games	Game	12	4.8	Reduction, Personalization, Liking, Expertise, Surface credibility, Real-world feel, Authority	7
22	CozZo . Food Inventory Manager	iOS	Sep 1, 2019	Food & Drink	Food WM	12	4	Reduction, Tailoring, Personalization, Rehearsal, Praise, Rewards, Reminders, Surface credibility, Real-world feel, Verifiability	10
23	CUI Curbside Collection	Android	August 1, 2019	Productivity	Regional waste disposal	27	4.3	Tailoring	1
24	Cumberland County Solid Waste	Android	August 1, 2019	Productivity	Regional waste disposal	125	4.4	Reduction, Tailoring, Personalization, Rehearsal, Praise, Rewards, Reminders, Surface credibility, Real-world feel, Verifiability	10
25	Cycled	Android	February 27, 2019	Social	Marketplace	13	4.6	Surface credibility	1
26	Don't Waste Waste	Android	September 29, 2018	House & Home	Regional waste disposal	7	4.1	Reminders, Authority, Verifiability	3
27	Dumpsters on Demand - RRO	iOS	Mar 23, 2018	Business	Commercial WM	2	5	Tailoring, Personalization, Rehearsal, Praise, Rewards, Reminders, Surface credibility, Real-world feel, Verifiability	9
28	Durham Region Waste	Android	August 1, 2019	Productivity	Regional waste disposal	2902	4.7	Personalization, Rehearsal	3

29	Eastern NS Waste Info	Android	August 1, 2019	Productivity	Regional waste dis-posal	62	4.9	Reduction, Tailoring, Personalization, Reminders, Sur-face credibility, Real-world feel, Verifiability	7
30	EasyWaste	Android	October 3, 2019	Business	Marketplace	5	5	Reduction, Rewards, Trustworthiness, Surface credi-bility, Real-world feel, Social facilitation	6
31	Eco360	Android	July 31, 2019	Productivity	Regional waste dis-posal	730	4.9	Reduction, Reminders, Surface credibility, Real-world feel, Verifiability	5
32	ECS e-Waste	Android	July 5, 2017	Business	Marketplace	49	4.1	Reduction, Tailoring, Personalization, Reminders, Sur-face credibility, Real-world feel, Verifiability	7
33	Edmonton Waste Wise	Android	August 1, 2019	Productivity	Regional waste dis-posal	296	4.5	Reduction	2
34	eevie - Eco Habit Tracker	iOS	Nov 4, 2019	Lifestyle	Personal tracking	4	3.3	Reduction, Personalization, Reminders, Real-world feel, Verifiability	5
35	Emralscleanup game	iOS	May 2, 2019	Lifestyle	Game	4	4	Surface credibility, Real-world feel, Social facilitation	3
36	Enviourenmental Studies Com-plete Guide (OFFLINE)	Android	October 13, 2017	Education	Education	103	4.6	Reduction, Tailoring, Reminders, Surface credibility, Real-world feel, Verifiability	6
37	Environment Challenge	Android	November 1, 2019	Lifestyle	Personal tracking	406	4.6	Reduction, Tailoring, Reminders, Surface credibility, Real-world feel, Verifiability	6
38	ENVIRONMENT PROTECTION CORPORATION	Android	August 11, 2019	Maps & Navigation	Biomedical WM	0	0	Personalization	1
39	Environmental Engineering	Android	April 11, 2019	Education	Education	8	2.5	Reduction	1
40	Environmental Engineering	Android	September 6, 2019	Education	Education	6	4.3	Simulation, Rewards, Liking, Social facilitation	4
41	Environmental Engineering 1	Android	April 24, 2019	Education	Education	151	4.1	Reduction, Tailoring, Personalization, Rehearsal, Praise, Rewards, Reminders, Surface credibility, Real-world feel	9
42	Environmental Engineering I	Android	January 9, 2018	Education	Education	129	4.2	Reduction, Tailoring, Personalization, Reminders, Sur-face credibility, Real-world feel	6
43	ENVIRONMENTAL ENGINEERING Quiz EXAM	Android	July 23, 2018	Education	Education	0	0	Reduction, Tailoring, Personalization, Rehearsal, Praise, Rewards, Reminders, Suggestion, Surface cred-ibility, Real-world feel	10
44	Environmental Science and Engi-neering	Android	March 12, 2018	Books & Reference	Education	33	4.4	Reduction, Personalization, Social facilitation	3
45	Environmental Studies	Android	March 29, 2019	Education	Education	10	4.1	Surface credibility, Real-world feel	3
46	ENVIRONMENTAL STUDIES NOTES	Android	June 9, 2018	Education	Education	51	4.1	Suggestion	1

47	Environmental Studies- Complete Reference Guide	Android	October 3, 2018	Education	Education	6	4.2	Reduction, Tailoring, Personalization, Rehearsal, Praise, Rewards, Reminders, Suggestion, Surface credibility, Real-world feel	10
48	ERA - Electronic Recycling	Android	August 13, 2014	Business	Waste collection	9	4.6	Reduction, Tailoring, Surface credibility, Real-world feel	4
49	Eswap	Android	April 15, 2018	Business	Commercial WM	5	5	Reduction, Tailoring, Personalization, Reminders, Suggestion, Surface credibility, Real-world feel, Verifiability	8
50	FoodHero - Fight Food Waste & Save Money	Android	October 2, 2019	Food & Drink	Food WM	156	4	Reduction, Tunneling, Personalization, Praise, Reminders, Suggestion, Liking, Social facilitation	9
51	FoodLess - Food Expiration Tracker	Android	November 4, 2019	Food & Drink	Food WM	12	4.4	Reduction, Tailoring, Personalization, Rehearsal, Praise, Rewards, Reminders, Suggestion, Surface credibility, Real-world feel, Verifiability	11
52	Foodprint	iOS	Sep 25, 2019	Food & Drink	Food WM	35	4.2	Reduction, Reminders, Real-world feel, Social facilitation	4
53	Foodro	iOS	Feb 13, 2019	Food & Drink	Food WM	0	0	Personalization, Simulation, Rewards, Liking, Social role	6
54	Fort Worth Garbage & Recycling	Android	August 1, 2019	Productivity	Regional waste disposal	715	4.8	Reduction, Tailoring, Personalization, Reminders, Surface credibility, Real-world feel, Verifiability	7
55	FreezerManager	iOS	Oct 9, 2019	Lifestyle	Regional waste disposal	0	0		0
56	Fridge Hero - Food Tracking	iOS	Aug 26, 2019	Food & Drink	Food WM	6	4.8	Real-world feel, Social facilitation	2
57	Fridgely	iOS	May 16, 2019	Food & Drink	Food WM	74	4.1	Reduction, Tailoring, Personalization, Reminders, Suggestion, Surface credibility, Real-world feel	7
58	Garbage and Recycling Day	Android	July 31, 2019	Productivity	Regional waste disposal	491	4.8	Surface credibility	1
59	Geev: The Zero Waste Solution	Android	November 4, 2019	Lifestyle	Food WM	8535	4.6	Surface credibility	1
60	Gibsons Waste Collection	Android	August 1, 2019	Productivity	Regional waste disposal	5	5	Personalization, Surface credibility, Social facilitation	3
61	Go Green Challenge	Android	June 14, 2019	Lifestyle	Personal tracking	77	4.2	Personalization, Praise, Rewards	3
62	Googreens	iOS		Lifestyle	Marketplace	0	0	Tunneling, Personalization, Praise, Reminders, Liking, Normative influence, Social facilitation	8
63	Halifax Recycles	Android	August 1, 2019	Productivity	Regional waste disposal	2717	4.6	Reduction, Tailoring, Personalization, Reminders, Trustworthiness, Surface credibility, Real-world feel, Normative influence	8

64	HCF Bio Medical Waste	Android	May 8, 2019	Business	Biomedical WM	15	4.7	Rehearsal, Rewards, Suggestion, Surface credibility, Social comparison, Social facilitation	6
65	Info on Environmental Protection Act 1990	Android	March 22, 2019	Books & Reference	Education	0	0	Reduction, Personalization, Reminders, Real-world feel, Social facilitation	7
66	InstaFresh	iOS	Nov 6, 2019	Food & Drink	Food WM	2	3	Reminders, Surface credibility, Real-world feel, Social facilitation	4
67	IVRI- Waste Management Guide App	Android	May 28, 2019	Education	Education	63	5	Reduction, Trustworthiness, Real-world feel	3
68	JK Medical Waste Management System	Android	August 11, 2019	Maps & Navigation	Biomedical WM	1	1	Personalization, Surface credibility, Social facilitation	3
69	KONET	Android	July 5, 2019	Business	Commercial WM	0	0	Reduction, Rewards, Reminders, Real-world feel, Verifiability	6
70	Lagos Waste Management Authority	Android	August 3, 2019	Lifestyle	Regional waste disposal	3	4.7	Personalization, Trustworthiness, Surface credibility, Real-world feel	4
71	Marck Waste	iOS	Sep 18, 2019	Business	Commercial WM	0	0	Reduction, Tailoring, Personalization, Rehearsal, Praise, Rewards, Reminders, Surface credibility, Real-world feel, Verifiability	10
72	Moira Shire Waste Info	Android	September 30, 2019	Tools	Regional waste disposal	5	4.1	Tunneling, Personalization, Suggestion, Real-world feel, Social facilitation	5
73	Mr.Fill Waste Manager	iOS	Oct 10, 2019	Utilities	Commercial WM	0	0	Reduction, Real-world feel, Verifiability	3
74	Murreys Disposal	Android	August 1, 2019	Productivity	Regional waste disposal	172	4.8	Reduction, Trustworthiness, Real-world feel	3
75	My City Cleaning - Waste Recycle Management	Android	October 26, 2018	Education	Game	124	3.9	Surface credibility, Social facilitation	2
76	My Little Plastic Footprint	iOS	Oct 1, 2019	Education	Plastic WM	2	4.5	Reduction, Real-world feel	2
77	My Waste	Android	May 15, 2019	Productivity	Regional waste disposal	5475	4.2	Reduction, Personalization, Reminders, Surface credibility, Real-world feel, Authority	6
78	MyACETTrashMN	iOS		Reference	Regional waste disposal	29	4.8	Reduction, Trustworthiness, Expertise, Real-world feel, Verifiability	5
79	NoWaste - Food Inventory List	iOS	Sep 10, 2019	Food & Drink	Food WM	100	4.3	Reduction	1
80	Ocean's Zero	Android	April 12, 2019	Lifestyle	Personal tracking	204	4.1	Reduction, Personalization	2
81	OLIO - Share more. Waste less.	Android	October 23, 2019	Food & Drink	Food WM	3027	3.5	Personalization, Reminders, Real-world feel	4

82	OneHalton	Android	November 8, 2018	Social	Regional waste disposal	29	2.8	Reduction, Tailoring, Reminders	4
83	Ottawa Collection Calendar	Android	August 1, 2019	Productivity	Regional waste disposal	2688	4.8	Reduction, Personalization, Reminders, Surface credibility, Real-world feel	5
84	Oxford County Wasteline	Android	August 1, 2019	Productivity	Regional waste disposal	285	4.7	Tunneling, Personalization, Suggestion, Real-world feel, Social facilitation	5
85	Peel Garbage Collection	Android	December 14, 2017	House & Home	Regional waste disposal	16	4.2	Reduction, Surface credibility	2
86	Peovica	Android	November 10, 2017	Business	Regional waste disposal	7	4.5	Reduction, Tailoring, Personalization, Reminders, Surface credibility, Real-world feel	6
87	Pick Pink	iOS	Apr 10, 2018	Utilities	Waste collection	4	4	Reduction, Tailoring, Personalization, Reminders, Surface credibility	5
88	Plastic waste recycling projects	Android	October 1, 2018	Entertainment	DIY projects	0	0	Reduction, Personalization, Reminders, Surface credibility, Real-world feel	5
89	Plutocalc Water and Wastewater	Android	September 8, 2018	Tools	Calculator	136	4.7	Reduction, Tailoring, Personalization, Reminders, Liking, Trustworthiness, Surface credibility, Real-world feel, Normative influence	10
90	Recycle Challenge - Fast, fun and educational!	Android	July 12, 2019	Casual	Game	5	4.9	Reduction, Personalization, Reminders, Real-world feel, Authority, Third-party endorsements, Social facilitation	7
91	Recycle Run	Android	June 3, 2015	Racing	Game	35	4.5	Personalization, Praise, Surface credibility, Cooperation	5
92	Recycle!	Android	November 16, 2018	Lifestyle	Regional waste disposal	20,264	4.2	Reduction, Tailoring, Personalization, Reminders, Surface credibility, Real-world feel	6
93	Recycling	Android	February 28, 2017	Entertainment	Education	15	2.9	Personalization, Surface credibility, Real-world feel	3
94	reGAIN app	iOS	May 10, 2019	Shopping	Cloth WM	74	4.7	Reduction, Rewards, Reminders, Surface credibility, Third-party endorsements, Social comparison	6
95	Region 6 NS Recycles	Android	July 31, 2019	Productivity	Regional waste disposal	18	4.8	Tunneling, Personalization, Suggestion, Real-world feel, Social facilitation	5
96	RePrint Footprint Tracker	iOS	Mar 19, 2019	Lifestyle	Personal tracking	20	4.6	Reduction, Tailoring, Personalization, Reminders, Surface credibility, Verifiability	6
97	RSC 8 Solid Waste	Android	August 1, 2019	Productivity	Regional waste disposal	69	4.8	Reduction, Tunneling, Tailoring, Personalization, Social learning, Social facilitation	7

98	SAFETY OFFICERS BLOG	Android	November 6, 2017	Education	Education	32	4.8	Simulation, Rehearsal, Surface credibility	4
99	Saskatoon Recycle & Waste	Android	August 1, 2019	Productivity	Regional waste disposal	405	4.7	Reduction, Personalization, Simulation, Reminders, Trustworthiness, Expertise, Surface credibility, Real-world feel, Social comparison, Normative influence, Social facilitation, Cooperation	12
100	Scrapo - Plastic Recycling Marketplace	Android	October 9, 2019	Business	Marketplace	170	3.8	Simulation, Praise, Suggestion, Liking, Surface credibility, Real-world feel	6
101	Segtech Bio Waste	Android	July 30, 2019	Business	Biomedical WM	0	0	Reduction, Personalization, Surface credibility, Real-world feel	4
102	Smart Plastic Recycling	Android	October 11, 2016	Lifestyle	DIY projects	15	3.8	Personalization, Simulation, Rehearsal, Praise, Rewards, Suggestion, Liking, Surface credibility, Social comparison, Cooperation	11
103	Smart Trivandrum	Android	October 5, 2019	Tools	Regional waste disposal	145	4.1	Reduction, Tunneling, Suggestion	4
104	Solid Waste Management	Android	December 3, 2016	Education	Education	0	2.5	Reduction, Personalization, Reminders, Suggestion, Expertise, Surface credibility, Real-world feel, Third-party endorsements, Verifiability, Cooperation	11
105	Solid Waste Management	Android	November 14, 2018	Social	Regional waste disposal	0	0	Reduction, Tailoring, Personalization, Rehearsal, Praise, Rewards, Surface credibility, Real-world feel, Verifiability	9
106	SOS Waste	Android	January 3, 2019	Productivity	Commercial WM	0	0	Reduction, Reminders	3
107	Start Recycle	Android	January 4, 2019	Business	Commercial WM	18	4.8	Reduction, Tailoring, Personalization, Reminders, Surface credibility, Real-world feel, Verifiability	7
108	Stay-Fresh	iOS	Jul 10, 2019	Food & Drink	Food WM	5	5	Reduction, Personalization, Expertise, Surface credibility, Real-world feel	6
109	Surrey Rethink Waste	Android	August 1, 2019	Productivity	Regional waste disposal	634	4.6	Reduction, Rehearsal, Praise, Rewards, Surface credibility, Verifiability	6
110	Sustainable Citizen App	iOS		Education	Regional waste disposal	0	0	Reduction, Tailoring, Personalization, Reminders, Surface credibility, Real-world feel	6
111	Tadweer	iOS	Feb 21, 2017	Business	Regional waste disposal	2	5	Reduction, Reminders, Real-world feel	4
112	Tadweer Recycling Game	iOS	Jan 2, 2017	Games	Game	0	0	Personalization, Praise, Rewards, Authority, Cooperation	6
113	TakaCycle: Free Waste Collection for Recycling.	Android	October 4, 2019	Productivity	Regional waste disposal	0	0	Personalization, Reminders, Surface credibility, Real-world feel, Authority, Third-party endorsements	6

114	The Environment Magazine	Android	April 18, 2019	News & Magazines	Magazine	6	4.3	Surface credibility	2
115	The SmartBin App	Android	January 15, 2019	Simulation	Game	0	0	Reduction, Personalization	2
116	TOwaste	Android	April 26, 2019	Lifestyle	Regional waste disposal	97	3.9	Reduction, Rewards, Trustworthiness, Surface credibility, Social learning, Social comparison, Social facilitation	8
117	Trash Rain	Android	April 3, 2018	Action	Game	0	0	Personalization, Rewards	3
118	Unwrapped UAE	Android	September 11, 2019	Lifestyle	Regional waste disposal	0	0	Personalization, Reminders, Trustworthiness	4
119	USA Waste Management	Android	February 8, 2019	Business	Commercial WM	0	0	Personalization, Rewards	2
120	VanCollect	Android	August 1, 2019	Productivity	Regional waste disposal	2029	4.7	Reduction, Reminders, Surface credibility	4
121	Waste & Recycling Expo Canada	Android	August 23, 2019	Productivity	Conference	0	0	Reduction, Personalization, Rewards, Suggestion, Surface credibility, Real-world feel	6
122	Waste Atlas	Android	February 9, 2015	Tools	Data collection	15	4.5	Tailoring, Personalization, Reminders, Liking, Surface credibility, Real-world feel, Social facilitation	8
123	Waste Less	Android	April 3, 2019	Tools	Regional waste disposal	158	4.1	Reduction, Personalization, Rewards, Reminders, Liking, Trustworthiness, Surface credibility, Real-world feel, Third-party endorsements, Social learning, Social comparison, Social facilitation	14
124	Waste Management	Android	September 7, 2015	Education	Education	2	4	Reduction, Rewards, Reminders, Suggestion, Liking, Surface credibility, Real-world feel, Social facilitation	9
125	Waste Management City Cleanup	iOS	June 1, 2019	Games	Game	0	0	Reduction, Personalization, Reminders, Surface credibility, Real-world feel, Verifiability	6
126	Waste Management Exam StudyToken	Android	September 30, 2019	Education	Education	0	0	Reduction, Tunneling, Tailoring, Personalization, Simulation, Praise, Reminders, Suggestion, Liking, Expertise, Surface credibility, Real-world feel, Social facilitation, Cooperation	15
127	Waste Management Review	Android	February 10, 2019	Books & Reference	Magazine	0	0	Reduction, Simulation, Praise, Reminders, Suggestion, Social comparison, Social facilitation	8
128	Waste No Food	Android	February 21, 2019	Social	Food WM	0	0	Reduction	2
129	Waste No More - Grocery, Shopping Lists & Home Inventory!	iOS	Feb 02, 2015	Productivity	Food WM	0	0	Reduction, Tailoring, Personalization, Reminders, Suggestion, Social facilitation	6
130	Waste Today	Android	September 9, 2019	Business	Magazine	0	0	Reduction, Personalization, Reminders, Suggestion	5

131	Waste Water Engineering Exam Quiz	Android	November 6, 2018	Education	Education	0	0	Reduction, Personalization, Suggestion, Surface credibility, Third-party endorsements	6
132	Waste Water Engineering Test Prep 2019 Ed	Android	May 9, 2019	Education	Education	0	0	Tailoring, Personalization	3
133	Waste Wise Greater Sudbury	Android	August 1, 2019	Productivity	Regional waste disposal	57	4.4	Reduction, Tunneling, Tailoring, Personalization, Simulation, Reminders, Suggestion, Liking, Trustworthiness, Expertise, Surface credibility, Real-world feel, Third-party endorsements, Verifiability, Social learning, Normative influence, Social facilitation, Cooperation	20
134	WasteCon 2018	Android	September 23, 2018	Events	Conference	0	0	Reduction, Tunneling, Tailoring, Personalization, Simulation, Praise, Rewards, Reminders, Suggestion, Liking, Trustworthiness, Expertise, Real-world feel	14
135	WasteConnect	Android	August 23, 2019	Productivity	Regional waste disposal	126	4.5	Reduction, Tailoring, Personalization, Reminders, Liking, Surface credibility, Social facilitation	8
136	Waterloo Garbage & Yard Waste	Android	April 15, 2019	Lifestyle	Regional waste disposal	17	3.7	Reduction, Tailoring, Suggestion, Similarity, Real-world feel	6
137	Wespack Client	iOS	Feb 14, 2018	Business	Commercial WM	0	0	Reduction, Personalization, Suggestion	4
138	What Goes Where	iOS	May 29, 2019	Reference	Regional waste disposal	48	4.7	Reduction, Personalization, Reminders, Social facilitation	5
139	Whitby Waste Buddy	Android	August 1, 2019	Productivity	Regional waste disposal	721	4.8	Reduction, Simulation, Rehearsal, Praise, Rewards, Reminders, Similarity, Authority, Social facilitation, Cooperation	11
140	Whitey Services	Android	February 5, 2018	Business	Commercial WM	0	0	Reduction, Tailoring, Personalization, Reminders, Suggestion, Trustworthiness, Real-world feel, Verifiability, Social facilitation	9
141	WM CartWise	iOS	Jun 6, 2019	Reference	Regional waste disposal	15	3.3	Reduction, Tunneling, Praise, Rewards, Suggestion, Real-world feel, Social facilitation	7
142	WMSBS	Android	August 14, 2019	Business	Commercial WM	6	4.3	Reduction, Personalization, Reminders, Suggestion	5
143	WRE	iOS	Oct 12, 2017	Utilities	AI aided waste sorting	0	0	Reduction, Tunneling, Simulation, Rewards, Suggestion, Social facilitation	7
144	Yo-Waste	Android	July 31, 2019	Business	Waste collection	0	0	Reduction, Personalization, Praise, Rewards, Reminders, Similarity, Liking, Surface credibility, Social facilitation, Cooperation	11

145	YWaste - Reduce food waste	Android	September 8, 2019	Food & Drink	Food WM	125	3	Reduction, Rewards, Social comparison	4
146	Zero Waste Objective	Android	August 27, 2017	Lifestyle	Personal tracking	70	3.8	Reduction, Tunneling, Suggestion, Real-world feel, Verifiability, Social facilitation	6
147	Zero-Waste - Avoid waste!	Android	November 10, 2019	Lifestyle	Personal tracking	27	4.3	Reduction, Personalization, Reminders, Surface credibility, Verifiability, Social facilitation	7
148	ZeroWasteHeroApp	Android	February 5, 2019	Lifestyle	Personal tracking	19	4.6	Personalization, Praise, Reminders, Suggestion, Cooperation	
